# Coping with Stress in Neoplastic Diseases

**DOI:** 10.3390/ijerph19159675

**Published:** 2022-08-05

**Authors:** Dominik Olejniczak, Paulina Mularczyk-Tomczewska, Krzysztof Klimiuk, Agata Olearczyk, Aleksandra Kielan, Anna Staniszewska, Karolina Osowiecka

**Affiliations:** 1 Department of Public Health, Faculty of Health Science, Medical University of Warsaw, 02-091 Warszawa, Poland; 2 Faculty of Medicine, Medical University of Gdańsk, 80-210 Gdansk, Poland; 3 Department of Experimental and Clinical Pharmacology, Medical University of Warsaw, 02-091 Warszawa, Poland; 4 Department of Psychology and Sociology of Health and Public Health, School of Public Health, University of Warmia and Mazury in Olsztyn, 10-719 Olsztyn, Poland

**Keywords:** neoplastic disease, chronic disease, stress, quality of life

## Abstract

Introduction: Disease-related stress is a common phenomenon. It also occurs in neoplastic diseases. Since physical and mental health are interrelated, it is important to make sure that treatment covers these two areas. Therefore, it is essential to learn how patients with neoplastic diseases can cope with stress. Materials and Methods: The respondents are 306 patients suffering from neoplastic diseases, associated in patient advocacy groups. The method is the Brief-COPE (Coping Orientation to Problems Experienced) questionnaire. Results: The following stress management strategies were most commonly adopted by the patients: acceptance (median 2.25; 25–75% IQR 2.0–3.0), active coping (median 2.0; 25–75% IQR (interquartile range) 1.5–2.0), planning (median 2.0; 25–75% IQR 2.0–2.0), emotional support (median 2.0; 25–75% IQR 1.5–2.0), instrumental support (median 2.0; 25–75% IQR 2.0–2.0), self-distraction (median 2.0; 25–75% IQR 1.5–3.0), and venting (median 2.0; 25–75% IQR 1.5–3.0). A decision to adopt a particular stress management strategy by patients with neoplastic diseases was highly affected by demographic factors (*p* < 0.05), such as sex, education, age, place of residence and employment. Conclusions: Teaching stress management strategies should be a part of the education process among patients with neoplastic diseases. Before or in the course of treatment, an oncology patient should be educated on the prevention of mental health disorders. The ability to cope with stress is one of the key competences for the course of neoplastic diseases and it can affect the treatment process. Stress management in chronic diseases, including neoplastic diseases, should be approached not only at the level of an individual person but also at the level of the health system as a whole.

## 1. Introduction

A diagnosis of a neoplastic disease often becomes a source of considerable stress for a patient and their family, which has been shown in many studies, e.g., by Sklar and Anisman [1]. Necessity to change one’s lifestyle and adapt to limitations imposed by the disease are just one of many after-effects and are frequently a heavy burden for one’s mental health, which has been investigated in many studies over the years, e.g., by Lissoni et al. [2]. Considering the above, it is essential to make education about stress management an integral part of the treatment process. Physical and mental health are interrelated, and, thus, better mental well-being might determine treatment effectiveness, e.g., through consequent participation in treatment procedures or maintaining an appropriate level of compliance or adherence. It has been shown that there is an interrelation between the location of cancer and the patient’s mental health condition [3].

It seems essential to monitor patient’s mental health condition in the course of treatment in order to promptly make an intervention, if necessary. An obvious recommendation on the treatment of patients with neoplastic diseases is to involve a psychologist or a psychiatrist in the therapeutic team. This solution allows for a more comprehensive patient care [3].

Currently, only few studies can be found in the global scientific literature on stress management in patients with neoplastic diseases. Patient guidebooks typically include information on external solutions (e.g., oncology rehabilitation, support groups, or psychological support). Certain studies have shown a positive influence of the task model on treatment and patient–doctor cooperation [3]. However, there is lack of studies concerning stress management strategies used by the patients with neoplastic diseases, especially during the COVID-19 pandemic.

The aim of the study was to identify the strategies of stress management used by cancer patients.

## 2. Materials and Methods

The study group consisted of 306 patients diagnosed with neoplastic diseases and associated in the patient advocacy groups, which are focused on advocacy for patients, survivors, and caregivers. Access to the study group was obtained from oncological organizations cooperating in the framework of national educational program for patients PACJENCI.PRO, the Academy of Patient Organization Development. The project was carried out by the Employers’ Union of Innovative Pharmaceutical Companies INFARMA and the Medical University of Warsaw, under the auspices of the Patients’ Rights Office. Inclusion criteria to the study were age ≥18 years old and cancer diagnosis.

In the study, the Brief-COPE standardized questionnaire was used [4]. It is a tool for examining adults, both healthy and ill. It consists of 28 statements that are assigned to 14 strategies (2 statements per strategy): active coping, planning, positive re-framing, acceptance, humor, religion, emotional support, instrumental support, self-distraction, denial, venting, substance use and behavioral disengagement. The respondent comments on each statement on a scale from 0 (I almost never do it) to 3 (I almost always do it). This method is typically used to measure one’s coping mechanisms, i.e., to assess typical ways of reacting to and experiencing highly stressful events.

The Polish version of the Brief-COPE questionnaire was validated by Juczyński and Ogińska-Bulik [4].

Reliability: Internal consistency reliability of the Polish version of Brief-COPE was established on the basis of a study conducted in 200 persons aged 25–60 years. Split-half reliability was 0.86 (Guttman’s coefficient of 0.87). Consistency is satisfactory for most scales.

Validity: Factor loadings of individual statements can be assumed as satisfactory. Diagnostic validity was assessed by correlating the results of Brief-COPE with the Mini-MAC scale, which is used to examine the strategy of coping with a neoplastic disease, and by forecasting the intensification of post-traumatic stress symptoms in mothers of children with leukemia.

Norms: Norms for university students and adults aged 20–65 years are expressed in means and standard deviations.

Use: The questionnaire is used to assess the ways of coping with stress. It was developed for scientific purposes but it can also be used for screenings and preventive tests, and to assess the effectiveness of therapeutic interactions.

The questionnaire was conducted using the CAWI method (computer-assisted web interview). It was published online as a Google Form. The results were collected automatically by the Form and then analyzed in a statistical program.

Consent of the Bioethics Committee of the Medical University of Warsaw was obtained for the research. Protocol number of the ethics is AKBE-161/2022. Patients were informed about the objective of the study. Each patient qualified for the studies received information that the collected results will be used only for scientific purposes. The participation in the study was voluntary.

### Statistical Analysis

Characteristics of the study group were analyzed using descriptive statistics: *n*, percentage, mean, SD (standard deviation), median, 25–75% IQR (interquartile range). The differences between the subgroups were analyzed with the Mann–Whitney test. The Spearman correlation coefficient was estimated to evaluate the relationship between age and different strategies of coping with stress. A *p*-value of <0.05 was considered to be significant. The analysis was conducted using Statistica, version 13 (http://statistica.io, accessed on 7 May 2022). TIBCO Software Inc., Krakow, Poland (2017).

## 3. Results

306 patients participated in the study, the majority of whom were women (83.3%). The mean age was 39 years. Half of the respondents lived in cities with more than 100,000 residents. Most respondents had university education and worked full-time (66.7%). A total of 83.3% of respondents were in a formal/informal relationship (Table 1).

**Figure 1:**
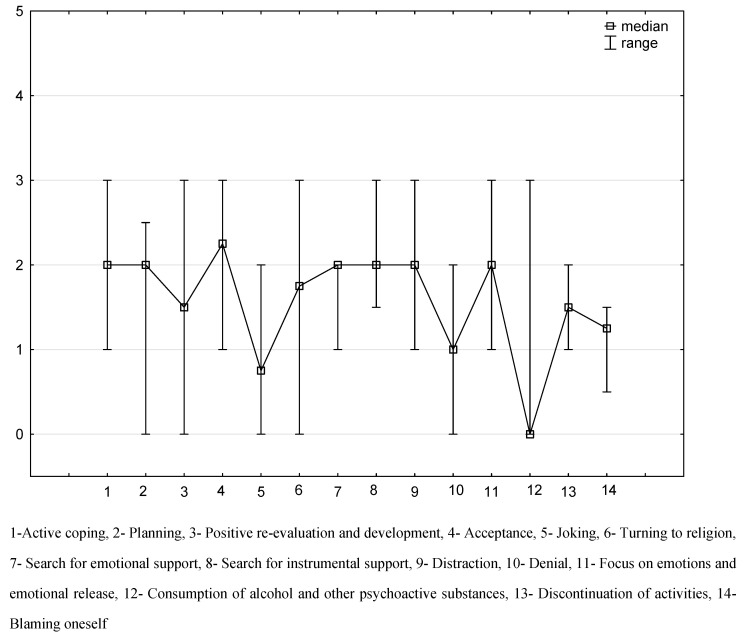
Stress management strategies.

The most common stress management strategies that were adopted by patients with neoplastic diseases in the course of oncological treatment were as follows: acceptance (median 2.25; 25–75% IQR 2.0–3.0), active coping (median 2.0; 25–75% IQR 1.5–2.0), planning (median 2.0; 25–75% IQR 2.0–2.0), emotional support (median 2.0; 25–75% IQR 1.5–2.0), instrumental support (median 2.0; 25–75% IQR 2.0–2.0), self-distraction (median 2.0; 25–75% IQR 1.5–3.0), and venting (median 2.0; 25–75% IQR 1.5–3.0). The least commonly adopted stress management strategies were substance use (median 0.0; 25–75% IQR 0.0–0.0), humor (median 0.75; 25–75% IQR 0.5–2.0) and denial (median 1.0; 25–75% IQR 0.5–1.5) (Figure 1).

The following demographic factors significantly affected the strategies of coping with stress among patients with neoplastic diseases: sex, education, age, place of residence, and employment.

Women used the following stress management strategies significantly more than men (*p* < 0.05): planning, active coping, positive re-framing, religion, discontinuation of activity, and substance use. Compared to women, men more frequently (*p* < 0.05) chose acceptance, emotional and instrumental support, self-distraction, venting, humor and blaming oneself (Table 2).

There is a positive correlation (*p* < 0.05) between the age of patients and the strategies of active coping, planning, shift to religion, emotional support, and blaming oneself. However, the older the patients get, the less frequently they choose the strategies of acceptance, humor, instrumental support, self-distraction, venting, substances use, and discontinuation of activity (*p* < 0.05) (Table 3).

Residents of cities with fewer than 100,000 residents statistically significantly more often choose emotional and instrumental support, and adopt the strategy of denial and blaming oneself (*p* < 0.05). Patients living in cities with more than 100,000 residents more frequently use the strategies of active coping, planning, positive re-framing, humor, religion, substance use and discontinuation of activity (*p* < 0.05) (Table 4).

Compared to respondents with secondary school education, patients with higher education significantly more often adopted the strategies of active coping, planning, positive re-framing, religion, and using alcohol or other psychoactive substances (*p* < 0.05). In turn, emotional and instrumental support, self-distraction, denial, venting, discontinuation of activity, and blaming oneself were more common in persons with secondary school education (*p* < 0.05) (Table 5).

Active coping, planning, positive re-framing, and substance use were more commonly adopted by the professionally active respondents, whereas the unemployed significantly more often sought emotional and instrumental support (*p* < 0.05) (Table 6).

### Limitation of the Study

The study was limited by the size of the sample and the selected group of respondents. The analysis included cancer patients associated in the patient advocacy groups.

## 4. Discussion

Cancer diagnosis is a traumatic event in patients’ life. Patients use different strategies to deal with distress, which could have impact on treatment compliance, psychological health and quality of cancer patients’ life. Psychological help is essential to identify stress management strategies and modify them. In our study, the most frequent strategy used by cancer patients was acceptance. Patients tend to focus on coping with stress through planning, active coping, self-distraction, venting and religion. Sabanciogullari and Yilmaz showed the positive relationship between positive religious-coping style and hope levels of cancer patients receiving chemotherapy [5]. Other authors also confirmed that turning to religion by patients could influence compliance to the disease, and treatment among cancer patients [6,7,8]. In our study, patients tended towards emotional and instrumental support. They should receive psychosocial help from health professionals. Only 21% of cancer patients in Poland received psychological support from psychologists and 4% from priests. Only 7% of cancer patients claimed that they received help from a social worker [9]. Rarely, patients tended to use stress management strategies such as humor, denial and blaming oneself. It is a positive phenomenon that destructive strategies, such as substance use, are rather uncommon in our study. This is a positive result considering other studies, e.g., a study by Esterbauer et al., which showed that chronically ill patients react to stress with the consumption of drugs and nicotine [10]. The results presented above indicate that a chronically ill person should be monitored and made secure against the use of psychoactive substances, as an irrational method of coping with the disease-related stress. This is also applicable to diseases other than neoplastic diseases. Importantly, many researchers, e.g., Alter, point out that focusing on pain, mood, mental stress and other problems related to quality of life can allow for effective treatment of symptoms and improvement in patient’s functioning, even in the case of late-stage neoplastic disease [11]. Currently, there are few studies available on the strategies of coping with stress in patients with neoplastic diseases. Patient guides typically contain information on external solutions (e.g., oncological rehabilitation, support groups, and psychological support). Certain studies show that there is a positive impact of the task model on treatment and patient–doctor cooperation. However, it is not known how many patients actually deal with stress in neoplastic diseases, or which stress management strategies are most commonly used by patients, especially during the COVID-19 epidemic [3].

Various demographic factors have a significant impact on the strategies of coping with stress in patients with neoplastic diseases, which has been confirmed by Esterbauer [10]. In this case, a multivariate analysis of covariance was used. Regarding age, men suffering from heart diseases showed higher values in the coping strategy tendency to flee. Women with heart complaints demonstrated significantly lower values in minimizing by comparison. In our study, patients with higher education, professionally active, women, younger and lived in bigger cities more often cope with stress using substances use. Identification of the risk group is important for introducing appropriate psychological support to improve their health, whereas lower education, unemployment, older age, living in smaller cities, and men are predisposing factors to seek emotional and/or instrumental support. This group should be monitored in the context of psychosocial support during oncological therapy. However, the quality of cancer patients’ life is very important.

The authors’ own study shows that compared to men, women significantly more often adopt the strategies of planning, active coping, positive re-framing, and religion. This is particularly important in light of the study by Ohaeri et al., who showed that women with breast cancer poorly adapt to the new situation [12]. One of the reasons behind slow adaptation is the lack of social acceptance for the disease, which is also emphasized by Tang et al. [13]. With regard to coping with stress, compared to women, men more often accept the situation, seek emotional and instrumental support, distract attention, vent, joke, and blame themselves. Considering the studies mentioned above, it is noticeable that most studies on stress in neoplastic diseases worldwide concern women, especially those with breast cancer.

The authors’ own study shows that age is a significant factor for stress management in illness: the older the patients get, the more frequently they adopt the strategies of active coping, planning, religion, searching for emotional support and blaming oneself. At the same time, the following strategies become less frequently applied: acceptance, humor, instrumental support, self-distraction, venting, substance use, and discontinuation of activity. Martins-Klein et al. noted that older adults less frequently reported cancer-related stress. [14] Whereas among women with breast cancer, older age was correlated with using maladaptive strategies, hopelessness and fatalism [15].

The authors’ own study shows that positive strategies, such as active coping, planning, positive re-framing, humor and religion are more frequently observed in patients living in cities with more than 100,000 residents. Unfortunately, patients in this group often adopt negative strategies, such as substance use and discontinuation of activity. These results are in line with the study by Amini-Tehrani et al., who directly presented the consequence of triggering the chain of identity changes (including distortion of body image), which ultimately result in full-scale psychological distress [16]. The thesis related to the distortion of body image was also confirmed in scientific studies, e.g., by Brandberg et al. [17]. Therefore, considering the conclusions of their own research, Schmid-Buchi et al. emphasized the importance of the needs of patients and their relatives, as well as psychological problems that require professional support even after the end of treatment. Continuous assessment of the disease symptoms and the needs of patients and their relatives constitutes the basis for targeted counseling and education [18]. In our study, patients living in smaller cities with fewer than 100,000 residents statistically significantly more often look for emotional and instrumental support and adopt the strategies of denial and blaming oneself. It is worth comparing the results presented above with the study by Tsaras et al., who indicated that considering a high risk of developing mental disorders, such as depression and anxiety, being a rural inhabitant or an unorthodox Christian with a high symptom burden may be a predictor of depression and anxiety in patients with breast cancer [19]. The role of cultural determinants of health are also emphasized by Onyedibe et al. or Herdman [20,21].

The authors’ own study shows that, compared with patients with secondary school education, patients with higher education significantly more often adopt the strategies of active coping, planning, positive re-framing, religion and substance use. In turn, acceptance, emotional and instrumental support, self-distraction, denial, venting, and disengagement prevail in respondents with secondary school education. Calderon et al. showed that breast cancer patients with lower educational level more often used hopelessness and cognitive avoidance [15]. These observations are in line with the studies by Onyedibe et al. or Herdman [20,21]. This allows for advancing a thesis that the quality of education can be a determinant of effectively coping with stress in neoplastic diseases. Therefore, it is clear that there is a need for an individual approach to the therapeutic process, also in terms of developing and implementing stress management strategies.

The authors’ own study shows that professionally active persons more often adopt not only the strategies of active coping, planning or positive re-framing, but also the strategy of substance use. Compared to the professionally active persons, the unemployed significantly more often sought emotional and instrumental support. There are few studies on this topic. Nevertheless, the available studies, e.g., by Taino et al., confirm the increase in the number of neoplastic diseases in the working population, which necessitates the creation of a therapeutic strategy for this group, including stress management strategies [22]. Pluta points out that professional work brings many benefits to the ill, but at the same time, it causes many difficulties. Both patients and employers are unaware of support options; intervention activities are rarely undertaken and the state’s aid is insufficient [23].

Distress related to cancer could lead to mental disorders. The prevalence of depression and anxiety was frequent among breast cancer women. From the meta-analysis, it was around 32% in the case of depression and around 42% of patients experienced anxiety [24,25]. Depression is one of the main health problems influencing lower survival rates [26,27]. A meta-analysis conducted by Wang et al. which included 282,203 breast cancer patients showed that depression was associated with higher risk of cancer recurrence (by 24%), and all-cause and cancer-specific mortality, respectively, by 30% and 29%. Anxiety as a second common psychiatric symptom was related to a 17% greater risk of cancer recurrence and 13% more risk of all-cause mortality [28]. Therefore, before or in the course of the treatment, each patient with a neoplastic disease should be taught how to prevent mental health disorders. The ability to cope with stress is one of the key competences for the treatment of neoplastic diseases. It has an impact on the effectiveness of the treatment, and, thus, it can improve the quality of life.

The issue of stress management in chronic diseases, e.g., neoplastic diseases, should be solved not only at the level of a “person”, but also at the level of a health system as a whole. The need for holistic patient care and the positive outcomes of holistic care have been known for a long time and they have been shown, e.g., by Becker, according to whom mental factors affect the immune system as a whole [29]. In addition, it has also been confirmed, e.g., by Popoola, that the course of a neoplastic disease predisposes depression [30]. This indicates anticipation of the occurrence of mental disorders in patients with neoplastic diseases and, thus, suggests the necessity of early preparation of effective solutions.

## 5. Conclusions

In our study, cancer patients mostly used stress management strategies: acceptance, active coping and planning. Although patients frequently accepted cancer disease, they needed emotional and instrumental support.

Each patient should be provided with appropriate psychological support at every stage of the disease, from diagnosis to the after-treatment period, because patient’s emotions and needs change at each stage. Different factors of sex, education, age, place of residence, and employment can influence stress-coping strategies used by cancer patients. The recognition and modification of destructive behaviors by psycho-oncologists can influence cancer patients’ well-being.

## Figures and Tables

**Table 1:** Target group characteristics.

All		*n* = 306	
Sex			
	woman	255	83.3
	man	51	16.7
Age [years]	mean±	39 ± 8.3	
Place of residence			
	city with less than 100.000 residents	153	50.0
	city with more than 100.000 residents	153	50.0
Education			
	secondary, vocational	102	33.3
	higher	204	66.7
Marital status			
	married, in a registered partnership	255	83.3
	divorced, separated	51	16.7
Employment			
	full-time worker	204	66.7
	unemployed	102	33.3

± Standard deviation.

**Table 2:** Stress management strategies according to sex.

	Women		Men		
Stress Management Strategies	Median	25–75% IQR	Median	25–75% IQR	*p*-Value
Active coping	2.00	(2–2)	1.00	(1–1)	<0.001
Planning	2.00	(2–2)	0.00	(0–0)	<0.001
Positive re-evaluation and development	2.00	(2–2.5)	0.00	(0–0)	<0.001
Acceptance	2.00	(2–2.5)	3.00	(3–3)	<0.001
Joking	0.50	(0.5–2)	1.00	(1–1)	0.02
Turning to religion	2.00	(1.5–2)	0.00	(0–0)	<0.001
Search for emotional support	2.00	(1.5–2)	2.00	(2–2)	<0.001
Search for instrumental support	2.00	(2–2)	3.00	(3–3)	<0.001
Distraction	2.00	(1.5–2)	3.00	(3–3)	<0.001
Denial	1.00	(0.5–1.5)	1.00	(1–1)	1.0
Focus on emotions and emotional release	2.00	(1.5–2)	3.00	(3–3)	<0.001
Consumption of alcohol or other psychoactive substances	0.00	(0–0)	0,00	(0–0)	0.02
Discontinuation of activity	1.50	(1–1.5)	1.50	(2–2)	0.02
Blaming oneself	1.00	(1.0–1.5)	1.50	(2–2)	<0.001

IQR—interquartile range.

**Table 3:** Stress management strategies according to age.

Stress Management Strategies	r	*p*-Value
Active coping	0.21	<0.05
Planning	0.17	<0.05
Positive re-evaluation and development	0.09	>0.05
Acceptance	−0.8	<0.05
Joking	−0.6	<0.05
Turning to religion	0.64	<0.05
Search for emotional support	0.44	<0.05
Search for instrumental support	−0.34	<0.05
Distraction	−0.77	<0.05
Denial	−0.06	>0.05
Focus on emotions and emotional release	−0.77	<0.05
Consumption of alcohol or other psychoactive substances	−0.39	<0.05
Discontinuation of activity	−0.49	<0.05
Blaming oneself	0.37	<0.05

r—correlation coefficient.

**Table 4:** Stress management strategies according to the place of residence.

	City with Less than 100,000 Residents		City with More than 100,000 Residents		
Stress Management Strategies	Median	25–75% IQR	Median	25–75% IQR	*p*-Value
Active coping	2.00	(1–2)	2.00	(1.5–3)	<0.001
Planning	2.00	(0–2)	2.00	(2–2.5)	<0.001
Positive re-evaluation and development	1.00	(0–2)	2.00	(0.5–3)	<0.001
Acceptance	2.00	(2–3)	2.50	(1–3)	1.00
Joking	0.50	(0.5–1)	2.00	(0–2)	<0.001
Turning to religion	1.50	(0–2)	2.00	(1–3)	<0.001
Search for emotional support	2.00	(2–2)	1.50	(1–2)	<0.001
Search for instrumental support	2.00	(2–3)	2.00	(1.5–2)	<0.001
Distraction	2.00	(1.5–3)	2.00	(1–3)	0.09
Denial	1.00	(1–1.5)	0.50	(0–2)	<0.001
Focus on emotions and emotional release	2.00	(1.5–3)	2.00	(1–3)	0.09
Consumption of alcohol or other psychoactive substances	0.00	(0–0)	0.00	(0–3)	<0.001
Discontinuation of activity	1.50	(1–1.5)	1.50	(1–2)	<0.001
Blaming oneself	1.50	(1.5–1.5)	1.00	(0.5–1)	<0.001

IQR—interquartile range.

**Table 5:** Stress management strategies according to education.

	Secondary, Vocational		Higher		
Stress Management Strategies	Median	25–75% IQR	Median	25–75% IQR	*p*-Value
Active coping	1.50	(1–2)	2.00	(1.75–2.5)	<0.001
Planning	1.00	(0–2)	2.00	(2–2.25)	<0.001
Positive re-evaluation and development	1.00	(0–2)	1.50	(0.75–2.5)	<0.001
Acceptance	2.50	(2–3)	2.25	(1.5–2.75)	<0.001
Joking	0.75	(0.5–1)	1.25	(0.25–2)	0.07
Turning to religion	0.75	(0–1.5)	2.00	(1.5–2.5)	<0.001
Search for emotional support	2.00	(2–2)	1.75	(1.25–2)	<0.001
Search for instrumental support	2.50	(2–3)	2.00	(1.75–2)	<0.001
Distraction	2.50	(2–3)	1.75	(1.25–2.5)	<0.001
Denial	1.25	(1–1.5)	0.75	(0.25–1.5)	<0.001
Focus on emotions and emotional release	2.50	(2–3)	1.75	(1.25–2.5)	<0.001
Consumption of alcohol or other psychoactive substances	0.00	(0–0)	0.00	(0–1.5)	<0.001
Discontinuation of activity	1.50	(1.5–1.5)	1.25	(1–1.75)	<0.001
Blaming oneself	1.50	(1.5–1.5)	1.00	(0.75–1.25)	<0.001

IQR—interquartile range.

**Table 6:** Stress management strategies according to employment.

	Full-Time Worker		Unemployed		
Stress Management Strategies	Median	25–75% IQR	Median	25–75% IQR	*p*-Value
Active coping	2.00	(1.75–2.5)	1.50	(1–2)	<0.001
Planning	2.00	(2–2.25)	1.00	(0–2)	<0.001
Positive re-evaluation and development	2.00	(1.25–2.50)	0.50	(0–1)	<0.001
Acceptance	2.25	(1.5–2.75)	2.50	(2–3)	<0.001
Joking	1.25	(0.25–2)	0.75	(0.5–1)	0.07
Turning to religion	1.75	(1.25–2.5)	1.00	(0–2)	<0.001
Search for emotional support	1.75	(1.25–2)	2.00	(2–2)	<0.001
Search for instrumental support	2.00	(1.75–2)	2.50	(2–3)	<0.001
Distraction	2.00	(1.5–2.5)	2.25	(1.5–3)	0.07
Denial	1.00	(0.25–1.75)	1.00	(1–1)	1.0
Focus on emotions and emotional release	2.00	(1.5–2.5)	2.25	(1.5–3)	0.07
Consumption of alcohol or other psychoactive substances	0.00	(0–15)	0.00	(0–0)	<0.001
Discontinuation of activity	1.50	(1.25–1.75)	1.25	(1–1.5)	<0.001
Blaming oneself	1.00	(0.75–1.25)	1.50	(1.5–1.5)	<0.001

IQR—interquartile range.

## Data Availability

Not applicable.
